# Epigenetic modulation as a therapeutic approach for pulmonary arterial hypertension

**DOI:** 10.1038/emm.2015.45

**Published:** 2015-07-31

**Authors:** Jun-Dae Kim, Aram Lee, Jihea Choi, Youngsook Park, Hyesoo Kang, Woochul Chang, Myeong-Sok Lee, Jongmin Kim

**Affiliations:** 1 Department of Internal Medicine, Yale Cardiovascular Research Center, Section of Cardiovascular Medicine, Yale University School of Medicine, New Haven, CT, USA; 2 Department of Life Systems, Sookmyung Women's University, Seoul, Korea; 3 Department of Biology Education, College of Education, Pusan National University, Busan, Korea

## Abstract

Pulmonary arterial hypertension (PAH) is a rare but progressive and currently incurable disease, which is characterized by vascular remodeling in association with muscularization of the arterioles, medial thickening and plexiform lesion formation. Despite our advanced understanding of the pathogenesis of PAH and the recent therapeutic advances, PAH still remains a fatal disease. In addition, the susceptibility to PAH has not yet been adequately explained. Much evidence points to the involvement of epigenetic changes in the pathogenesis of a number of human diseases including cancer, peripheral hypertension and asthma. The knowledge gained from the epigenetic study of various human diseases can also be applied to PAH. Thus, the pursuit of novel therapeutic targets via understanding the epigenetic alterations involved in the pathogenesis of PAH, such as DNA methylation, histone modification and microRNA, might be an attractive therapeutic avenue for the development of a novel and more effective treatment. This review provides a general overview of the current advances in epigenetics associated with PAH, and discusses the potential for improved treatment through understanding the role of epigenetics in the development of PAH.

## Introduction

Pulmonary hypertension (PH) is a disorder in the lung vasculatures including the pulmonary artery, pulmonary vein or pulmonary capillaries, resulting in an increase of blood pressure followed by heart failure.^1^ After the clinical classification of PH into primary and secondary PH at the first meeting held by the World Health Organization (WHO) in 1973, the categories of PH were continuously subdivided more precisely, until reestablishment according to the presence of the identified causes at the 5th World Symposium of Pulmonary Hypertension held in Nice, France, in 2013. The recent updated classification of PH presents five WHO groups as follows: (i) WHO group 1, pulmonary arterial hypertension (PAH); (ii) WHO group 2, pulmonary hypertension due to left heart disease; (iii) WHO group 3, pulmonary hypertension due to lung diseases and/or hypoxia; (iv) WHO group 4, chronic thromboembolic pulmonary hypertension; and (v) WHO group 5, pulmonary hypertension with unclear multifactorial mechanisms. Each group was also further subdivided by its genetic or pathological causes.^2^ PAH, the WHO group 1, is a disorder of the pulmonary arterioles, resulting in increased blood pressure followed by right ventricular heart failure, and characterized by the absence of the common causes of PH, which include chronic liver and thromboembolic diseases. The pathogenic events of PAH arise from the hyperproliferation of pulmonary vascular cells, such as pulmonary artery endothelial cells (PAECs) and pulmonary artery smooth muscle cells (PASMCs), which in turn causes neointima formation in the small pulmonary arteries.^3^ Although rare, occurring at only 2.4–7.6 cases per million per year, PAH is a progressive disease leading to an incident mortality rate of ~15% within 1 year of diagnosis. Moreover, the mortality rate of PAH was reported in 2012, to be 51% within 7 years of diagnosis.^4, 5^

**Figure 1:**
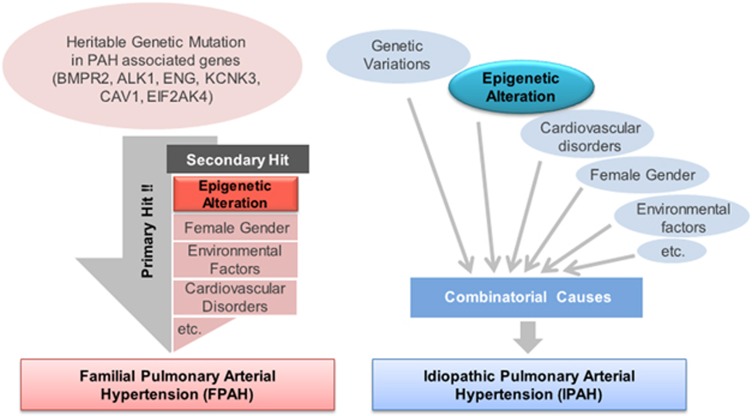
Proposed multifactorial pathogenesis of pulmonary arterial hypertension (PAH). This figure presents the complex nature of heritable PAH (HPAH) and idiopathic PAH (IPAH). In the case of HPAH, the major driver ‘primary hit' maybe genetic mutation of HPA-associated genes. In many PAH patients, unknown or undetectable ‘secondary hit' mechanisms such as epigenetic alteration, gender and other cardiovascular anomalies, as well as environmental factors, might cooperate in the progression of HPAH. Commonly, IPAH is caused by the combination effect of multiple cues such as non-heritable genetic or epigenetic variations, as well as environmental statuses.

PAH is a complex disease with multiple etiologies and may be mediated by the interplay of genetic background, epigenetic changes and pathobiological environmental factors, which explains the great variability in susceptibility^6^ (Figure 1). Therefore, the defining molecular mechanisms involved in the pathogenesis of PAH may arise from various aspects due to the multiple etiologies and disease heterogeneity. Emerging evidence has demonstrated the importance of epigenetics in the pathogenesis of PAH.^6, 7, 8, 9^ Epigenetics is defined as all heritable changes in gene expression that are not related to changes in the underlying DNA sequence.^10^ To date, the cell-signaling abnormalities, and environmental and genetic mechanisms involved in PAH pathogenesis, have been well studied. However, despite advances in epigenetics technology such as genome-scale DNA methylation analysis, few studies have yet been performed on the epigenetics associated with PAH pathogenesis. The three main types of epigenetic regulation are DNA methylation, histone modification and microRNA (miRNA).^11^ Although many miRNAs associated with PAH have been elucidated, the involvement of epigenetic regulation via methylation and histone modification in the pathogenesis of PAH remains in critical need of investigation. Our efforts for understanding the initiation and progression of PAH via epigenetics research may provide new insights to identify novel targets for treatment. This review will introduce the current understanding of the epigenetics associated with PAH pathobiology and discuss the possible epigenetic modulations involved in progression of PAH.

## Major mechanisms of epigenetic regulation

### DNA methylation

Although most epigenetic changes are highly dynamic, depending on the cellular status, DNA methylation is comparably stable and can even be inherited by daughter cells. In the genome, methyl groups can be added by covalent interaction to adenosine or cytosine DNA nucleotides through the enzyme DNA methyltransferase (DNMT).^12, 13^ DNA methylation and demethylation processes govern diverse biological conditions, involving genomic imprinting, cellular differentiation, organ morphogenesis, cell reprogramming, X-chromosome inactivation, RNA splicing, transposon silencing and DNA repair.^14^ The CpG island, a region with a specific base sequence commonly located in gene promoters, can undergo particularly high rates of methylation, which is considered as a sign of genetic repression. Mechanistically, DNA methylation achieves transcriptional repression through three modes of action as follows: (1) direct masking of the binding site of transcription factors, caused by nucleosome compaction; (2) recruitment of transcriptional repressors; and (3) cross-interaction with histone modification mechanisms.^15, 16^ Conversely, the promoter regions of most active genes appear to be unmethylated (or hypomethylated). For example, oncogenes are generally hypermethylated, whereas tumor suppressor genes are unmethylated in cells with normal cellular status; this epigenetic state is reversed in the progression of cancer.^17^ Consequently, the detection of global changes in DNA methylation can be considered as a hallmark of cancer pathogenesis.^18, 19^ During embryonic development, organogenesis and general pathogenesis, functional genes can also be controlled spatiotemporally by DNA methylation mechanisms. Moreover, changes of the DNA methylation state are known to have close relations to a variety of human diseases and disorders including cancer, diabetes, immune disorders, cardiovascular diseases and cerebral ischemia, as well as genetically imprinted disorders, suggesting the strong possibility for therapeutic applications of this epigenetic mechanism.^20^ In such diseases, detection of the epigenome state through analysis of the global DNA methylation state might be important, as epigenetic changes can be considered as biomarkers for interpretation of pathogenic status. The fact that the alteration of DNA methylation by environmental input can be observed is a very interesting biological phenomenon. In addition to genetic cues of pathogenic abnormality, this can lead to a unique synergetic condition through accumulation of hyper- or hypo-DNA methylation, causing it to be regarded as a ‘second hit' in many disease conditions. Alcohol, tobacco, cocaine and many drugs are capable of modifying the global DNA methylation in target genomic regions of sensitive organs, especially in conditions of pregnancy.^21, 22^

### Histone modification

Double-stranded DNA is highly packed with incorporation of histones and is organized as the nucleosome to build chromatin in eukaryotic cell nuclei. In nature, five histone families, H1/H5, H2A, H2B, H3 and H4, were found to be incorporated into chromatin. Although the H1/H5 histone family has the role of a linker, the other histones function to form a core structure with the DNA strand. In biological events, the posttranscriptional modification of these histones is related to diverse functional aspects including the regulation of gene transcription, DNA repair process, chromosome condensation and meiosis, as well as genetic imprinting.^23, 24^ Similar to other proteins, histones are diversely modified by posttranslational modification through the following mechanisms: methylation, acetylation, ubiquitination, SUMOylation, phosphorylation and ADP-ribosylation. These constitute the complex ‘Histone Code,' which is determined by the combinational modifications of histone.^25, 26, 27^ In particular, histone methylation and acetylation are closely linked with transcriptional gene regulation, which is regarded as one of the major epigenetic events and is related with many pathological conditions in the progression of cardiovascular disease, including PAH.^9, 26^ Many of the lysine (K) residues of histone H3 such as H3K4, H3K9, H3K27, H3K36 and H3K79 are commonly methylated by adding one, two or three methyl groups, respectively. Although the methylation of histone H3K9 and H3K27 is thought to be a code for transcriptional repression, the methylation of histone H3K4, H3K36 and H3K79 is able to induce gene transcription.^28^ A variety of histone methyltransferases and histone demethylases are involved in controlling the state of histone methylation. In addition to histone methylation, the acetylation of histone H3, especially of H3K9, H3K14 and H3K27, can also function as an active transcriptional code. These biological processes are governed by histone acetyltransferase and histone deacetylase (HDAC).^29, 30^ Specifically, HDAC removes the acetyl groups from lysine residues on the histone, causing chromatin to become tightly packaged, and represses gene expression. In humans, 18 HDACs within 4 classes have been discovered, including class I, IIa, IIb, III and IV. Except for class III HDACs, which are known as sirtuins (SIRT1-7), the other three classes of HDACs are zinc-dependent enzymes, which are commonly targets of small chemical HDAC inhibitors.^31, 32^ Recent evidence has suggested that inflammation widely contributes to cardiovascular pathogenesis, including the pathogenesis of PAH.^33^ Importantly, treatment with HDAC inhibitors at a low dose can attenuate the inflammatory response in chronic cardiovascular conditions, providing the potential functional mechanism of HDAC inhibitors in therapeutic treatment.^34^

### MicroRNAs

miRNAs are endogenous, short-length (20–24 nucleotides), noncoding RNAs that are involved in the posttranscriptional fine-tuning of gene expression, typically through binding to the 3′-untranslated region, to affect the stability and translation of target mRNAs. miRNAs are expressed in multicellular organisms and are highly conserved between species.^35^ As one miRNA has the capacity to target multiple mRNAs, elaborate regulation of miRNA expression is fundamental to maintaining homeostasis in living organisms. Accordingly, altered expression of miRNAs can cause pathogenic conditions such as PAH.^35^ In addition, emerging studies have shown that epigenetic modifications by DNA methylation and histone modification can also regulate the expression of miRNAs in a transcriptional manner, suggesting the therapeutic potential of HDAC and DNMT modulators for regulating miRNA expression for the treatment of PAH.^9, 36^

## Epigenetic alterations in PAH

### DNA methylation and histone modification in PAH

Given the exacerbated severity of PAH by the interplay of complex genetic and/or epigenetic changes, identification of novel therapeutic avenues via investigation of the epigenetic mechanisms involved in the pathogenesis of PAH might be of growing interest. However, few studies have identified the role of epigenetic modifications such as altered DNA methylation and histone modification in association with PAH pathogenesis.^7, 8, 9^

Superoxide dismutase-2 (SOD2) is a member of the iron/manganese SOD family, which catalyzes the dismutation of superoxide into hydrogen peroxide and diatomic oxygen, and has a critical role in vascular functions.^37^ Several lines of study have implicated SOD2 in the development of PAH.^8, 38^ One study showed that adenovirus-mediated gene transfer of SOD to the lung ameliorated monocrotaline-induced PH in rats, suggesting the involvement of increased oxidative stress in the pathogenesis of PAH and the therapeutic potential of antioxidants for treatment.^38^ Epigenetic changes by histone modification are a key mechanism for regulation of cell proliferation and survival. The first demonstration of aberrant epigenetic changes in PAH pathogenesis showed no mutation in the *SOD2* gene, whereas tissue-specific, methylation-induced SOD2 deficiency increased the proliferation and decreased the apoptosis of PASMC, while also impairing redox signaling. Conversely, SOD augmentation restores experimental PAH, suggesting therapeutic benefits of epigenetic modification.^8^

Zhao *et al.*^7^ also demonstrated that epigenetic modifications, through histone acetylation, are implicated in the development of PAH. The levels of HDAC1 and HDAC5 were higher in lungs from both PAH patients and a PH rat model than in control groups, and HDAC inhibitors were found to exert antiproliferative and anti-inflammatory effects on vascular cells. The authors also demonstrated that HDAC inhibitors, including suberoylanilide hydroxamic acid and vorinostat, ameliorate the phenotype of PH rat models, indicating that increased HDAC activity leads to the pathological condition of PH.^7^ Another investigation by Wang *et al.*^36^ showed that miR-124 has an important role in maintaining homeostasis in fibroblasts and is involved in the pathogenesis of PAH. miR-124 significantly inhibits proliferation, migration and expression of monocyte chemotactic protein-1 in pulmonary vascular fibroblasts. In addition, the expression of miR-124 is significantly decreased in the fibroblasts of patients with PAH. Interestingly, the decrease in miR-124 was restored by treatment with HDAC inhibitors, but not by 5-aza-deoxycytidine in hypertensive fibroblasts.^36^ These studies suggest therapeutic potential for HDACs inhibitors in the treatment of PAH.

Myocyte enhancer factor 2 (MEF2) is a family of transcription factors, which are known to have an important role in control of the expression of genes involved in cellular differentiation and embryonic development. There are four members of the MEF2 family: MEF2A, MEF2B, MEF2C and MEF2D. Among them, MEF2A and MEF2C are highly expressed in endothelial cells. Endothelial-cell-specific MEF2C-deficient mice showed reduced retinal vessel loss and decreased endothelial apoptosis, suggesting that MEF2 is a key endothelial homeostatic transcription factor in the vasculature.^39, 40, 41, 42^ In light of the function of MEF2 in endothelial cells, the most recent study by Kim *et al.*^9^ found a novel role for the transcription factor MEF2 in maintaining homeostasis in the pulmonary vasculature. MEF2 activity was also found to be impaired in PAH PAECs through excess nuclear accumulation of HDAC4 and HDAC5. The impaired MEF2 activity leads to the downregulation of target genes involved in pulmonary vascular homeostasis, including miR-424 and 503, connexins 37 and 40, and Krűppel-like factors 2 and 4 (Kim *et al.*^9^). Especially, miR-424 and 503 have been revealed as key miRNAs in maintaining homeostasis, the disruption of which lead to the pathogenesis of PAH.^43^ The authors also demonstrated that selective, pharmacological inhibition of class IIa HDACs using MC1568 restored the impaired MEF2 activity in PAH PAECs and rescued the experimental monocrotaline and SU-5416/hypoxia (SUGEN) PH models, while restoring targets such as miR-424 and 503 (Kim *et al.*^9^). The reports described above provide direct evidence of the role of epigenetics in PAH pathogenesis, contributing to the vascular pathology of PAH.

### miRNAs in PAH

**Figure 2:**
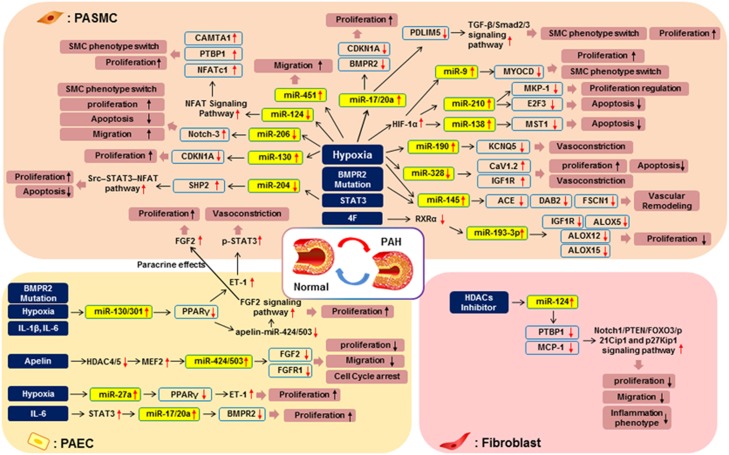
MicroRNA (miRNA) regulatory pathways implicated in the pathogenesis of pulmonary arterial hypertension (PAH). This figure provides an overview of the signaling pathways regulated by miRNAs, which are involved in the pathogenesis of PAH. Aberrant expression of miRNAs in pulmonary vascular cells such as pulmonary artery endothelial cells (PAECs), pulmonary artery smooth muscle cells (PASMCs) and fibroblasts by pathological factors leads to altered signaling pathways and contributes to the pathogenesis of PAH.

As PAH is a complex disease with multiple etiologies, miRNAs may be key candidates for more effective treatment, due to their capacity to coordinately regulate various signaling pathways associated with PAH such as bone morphogenetic protein (BMP) signaling, apelin (APLN) and apelin receptor (APLNR) signaling and hypoxia-related signaling, via the targeting of multiple mRNAs.^1, 44, 45, 46^ Thus, the abnormal expression and dysregulation of miRNAs contributes to the pathogenesis of PAH. A significant amount of study has been devoted to elucidating the roles of miRNAs, as one epigenetic mechanism involved in the pathogenesis of PAH, through which many altered miRNAs have been identified.^1, 35^ The PAH-related miRNAs and signaling mechanisms, including their target mRNAs, are summarized in Table 1 and Figure 2.

## Possible epigenetic alteration of genes associated with PAH

### Components of BMP signaling

BMP signaling is a part of the transforming growth factor (TGF)-β superfamily, which consists of TGF-β, BMP, activin and growth and differentiation factor (GDF) signaling.^47, 48^ The recent circumstantial evidence suggests that BMP signals are involved in the development of endothelium and maintenance of blood vessel homeostasis.^49, 50, 51^ In particular, several human genetic mutations have been closely linked with pathogenic conditions related to the cardiovascular system such as hereditary hemorrhagic telangiectasia and PH.^52, 53, 54^ The following section will discuss the potential possibility of epigenetic regulation of the major components involved in the BMP signaling cascade and the subsequent influence on PAH progression.

#### BMP type I receptors

BMP type I receptor (BMPR1) directly binds to BMP ligands such as BMP2, 4, 6, 9 and 10, and is activated by phosphorylation through subsequent formation of tetraheteromeric complexes with BMP receptor type II (BMPR2). In genetic screening experiments in PH (or PAH) patients, mutations in *BMPR1* genes, including activin A receptor type II-like 1 (*ACVRL1/ALK1*) and BMP receptor type-1B (*BMPR1B/ALK6*), were identified to be involved in predisposal to PAH.^55, 56^ Moreover, the downregulation of BMP receptor type-1A (BMPR1A/ALK3), mediated by angiopoietin-1 signaling in the lung tissue, was suggested to be a cause of nonfamilial PH.^57^ Although direct evidence of epigenetic regulation of the *BMPR1* genes has not yet been finely addressed in cardiovascular pathogenesis, there may be considerable functional contributions of epigenetic mechanisms to these genes. For instance, the expression of ACVRL1/ALK1, which is dominantly expressed in endothelial cells, is controlled by CpG island methylation, mediated by transcription factor Sp1 on the *ACVRL1/ALK1* promoter region, suggesting possible involvement of the epigenetic mechanism of ACVRL1/ALK1 in PAH.^58^ In recent times, miR-656 was reported to directly target BMPR1A/ALK3 in glioma cell lines.^59^ In the case of *BMPR1B/ALK6*, it was suggested that the expression of this gene can be tightly regulated through the DNA methylation status of the species-conserved 5′-CpG island in the promoter region. The hypermethylation of this region silences BMPR1B expression in human glioblastoma tumor-initiating cells and results in inhibition of normal cell differentiation and subsequent tumorigenicity. Moreover, treatment with a demethylation agent such as 5-aza-2′-deoxycytidine or genetic inhibition of enhancer of zeste homolog 2, which functions in the methylation of CpG islands by recruiting DNMT, restored the expression of BMPR1B in human glioblastoma tumor-initiating cells.^60^ Collectively, these epigenetic regulations of *BMPR1* genes may also be involved in PAH pathogenesis, and require further elucidation, as these biological phenomena have not yet been described in the cardiovascular system.

#### BMP receptor type II

The unique type II serine/threonine kinase receptors in components of the TGF-β superfamily, involving TGF-β, BMP and activin signaling, determine the downstream signaling cascade. BMPR2 has a unique signaling capacity only for BMP signal transduction, chiefly relayed by SMAD1, 5 and 8 (Ehrlich *et al.*^47^). Around 70% of familial PAH patients were found to have heritable genetic mutations of *BMPR2*. Moreover, genetic alteration of *BMPR2* was found in about 10% to 40% of idiopathic PAH (IPAH) patients.^61, 62^ However, the potential susceptibility to genetic alteration of *BMPR2* in heritable PAH and IPAH is relatively low, as ~20% of individuals have heritable mutations, suggesting incomplete penetrance of PAH and the requirement for another secondary cue to promote development of PAH.^63^ The germline mutation of *BMPR2* is regarded as the most common causative factor and can be targeted for primary therapeutic application in advance. Currently, the involvement of epigenetic alteration or regulation of BMPR2 in PAH progression has not been clearly defined; however, there are several examples of the involvement of epigenetic regulation of BMPR2 in various pathological conditions including cardiovascular disorders. For example, miR-17-5p and miR-20a were reported to be closely related to BMPR2 expression in PH pathogenesis, having a role in BMPR2 downregulation in PAEC and PASMC^64, 65^ (Figure 2). On the other hand, the involvement of other epigenetic regulations such as histone modification and DNA methylation of *BMPR2* are largely unknown and little have been suggested. In recent times, the CpG island on the *BMPR2* promoter was found to be hypermethylated in scleroderma endothelial cells, whereas treatment with a DNMT inhibitor and/or HDAC inhibitor reversed the enhanced apoptosis of the cells, illuminating the possible contribution of abnormal DNA methylation in scleroderma pathogenesis.^66^ These epigenetic contributions might also have a relation to other cardiovascular disorders, including PAH, and should be addressed further in PAEC and PASMC beds, to reveal possible roles in PAH.

#### Endoglin: co-receptor of TGF-β/BMP signaling

Although endoglin was originally identified as a TGF-β receptor, it is now regarded as a co-receptor for TGF-β and BMP signaling. In particular, the receptor activity of ALK1, which dominantly binds with the BMP9 or 10 ligands, can be modified by the presence or absence of endoglin. In human cardiovascular pathogenesis, *endoglin* has been identified as the causal gene for hereditary hemorrhagic telangiectasia type I, classified by the clinical presence of recurrent epistaxis and spontaneous arterial venous malformation, sometimes followed by PH or PAH.^67^ Several epigenetic mechanisms regulating the expression of endoglin were reported in tumor conditions. For example, endoglin was reported to be critically downregulated in numerous esophageal squamous cell carcinoma tissues, due to promoter hypermethylation of the *endoglin* locus. Treatment with a demethylation agent restored the endoglin expression in carcinoma cell lines, suggesting the potential epigenetic regulation of the *endoglin* promoter.^68^ Moreover, several miRNAs are known to target endoglin in endothelial cells or cardiac myocytes. In particular, miR-208a repressed endoglin expression in the heart, whereas miR-370 was negatively correlated with endoglin expression in endometrioid ovarian cancer cells.^69, 70^

#### SMADs: main mediators of BMP signaling

Several receptor-mediated SMADs such as SMAD1, 5, 8 or 9 and a common-mediator SMAD, SMAD4, are involved in canonical BMP signal transduction in various biological processes including the development and homeostasis of the cardiovascular system. Some functional and genetic involvements of these signaling mediators have been reported. The truncating mutation of *SMAD9* was identified in familial PAH patients, and genetic variants in SMAD1 and SMAD4 were identified as a causative factor for IPAH.^71, 72^ Interestingly, the pathological results from a *SMAD9* knockout mouse model strongly support the functional involvement of *SMAD* genes in the progression of human PAH.^56^ It was demonstrated that the translation of *SMAD* genes is epigenetically modified by multiple sets of miRNAs in various cell types such as endothelial and vascular smooth muscle cells, as well as mesenchymal stem cells, for bone differentiation. For example, miR-4448, -4708 and -4773 target SMAD1 and SMAD4 transcripts, to induce osteoblast differentiation from mesenchymal stem cells.^73^ Moreover, miR-26a, -30b and -205 are more closely involved in the cardiovascular system. miR-30b represses SMAD1 expression in aortic valve interstitial cells and its reduction might lead to calcific aortic valve disease.^74^ Several reports in the literature have suggested that SMAD1 and SMAD4 are common targets of miR-205 and -26a in endothelial and vascular smooth muscle cells, respectively, which are major contributors to lung homeostasis.^75, 76^ The translation of SMAD5 can be repressed by miR-155 and its similar sequence homolog, miR-K12-11, which is encoded by Kaposi's sarcoma-associated herpesvirus.^77, 78^ Although the contribution of miRNAs targeting multiple *SMAD* genes in PAH has not yet been evaluated clearly, the possible involvement may be considerable.

#### Caveolin-1

Caveolin-1 is a membrane protein, which has the role of forming caveolae, and mainly functions in the endocytosis.^79^ In recent times, it was discovered that two frameshift mutations, a c.474delA and c.473delC, in the highly conserved C terminus amino acid sequence of caveolin-1 are related to familial PAH and IPAH.^63^ Interestingly, several reports suggest that the caveolae is able to control multiple signaling inputs in the endothelium, including BMPR2 and nitric oxide (NO) signaling.^80, 81^ Contributions of epigenetic DNA methylation to the expression of caveolin-1 have been suggested in adipocyte differentiation and soft tissue sarcoma. In particular, methylation of the promoter, exon1 and first intron region of caveolin-1 is extensively removed, inducing strong expression of caveolin-1 during adipocyte differentiation.^82^ Moreover, epigenetic regulation of caveolin-1 by multiple miRNAs such as a miR-133a, 802, 103, 107, 199a-3p, 199a-5p, 203 and 124 has been reported in diverse biological environments, including in insulin signaling, tumor cell proliferation and migration, and kidney homeostasis.^83, 84, 85, 86, 87^ Although the direct involvement of epigenetic regulation of caveolin-1 has not been revealed in the cardiovascular system, some of the suggested epigenetic mechanisms listed above may also be involved in cardiovascular pathogenesis, including PAH.

#### KCNK3 potassium channel

KCNK3 (Potassium channel subfamily K member 3) is a two-pore potassium channel majorly expressed in PASMC. In hypoxic conditions, KCNK3 is responsible for the control of membrane potential homeostasis. Currently, six cases of missense mutation (T8K, G97R, E182K, Y192C, G203D and V221L) have been reported in familial PAH and IPAH patients.^88, 89^ The mechanisms for epigenetic control of this gene in the cardiovascular system have not yet been elucidated, as this genetic alteration was only recently identified as a pathogenic cue of PAH.

## Current therapies for PH

The current therapies for PAH patients commonly aim to reverse the imbalance of pulmonary vasoactive mediators. NO and prostacyclin are known vasodilators, whereas endothelin-1 (ET-1) and thromboxane A2 are known to function for vasoconstriction in PAEC and PASMC. Prostacyclin, a lipid produced by endothelial cells, causes vasodilatation and prevents the coagulation of platelets. It was reported in the 1990s that intravenous therapy with epoprostenol, a synthetic prostacyclin derivative, in combination with conventional therapies such as treatment with anticoagulants, cardiac glycosides and supplemental oxygen can reverse the severe progression of IPAH. Moreover, continuous infusion therapy with epoprostenol was found to be effective for several types of PAH patients, including those with PAH caused by systemic sclerosis or other connective tissue disease. To attenuate the progression of PAH, prostacyclin analogs are also available for inhalation as an aerosol formulation, or treatment as an orally active sustained release form.^90, 91^ Currently, many medical research groups are developing application methods for prostacyclin treatment, most of which are in clinical trials for multiple types of PAH patients including IPAH, connective tissue disease-associated PAH, HIV-associated PAH and congenital heart disease-associated PAH. Another target for PAH treatment is the ET receptor.^91^ ET is produced by endothelial cells and has a role in smooth muscle contraction. Mechanistically, ET binds to ET receptor type A (ET-A) and type B. Interestingly, the signaling output through ET receptor type B can cause a decline in the effects of ET-A, inducing mitogenic effects and vasoconstriction of vascular smooth muscle cells.^92^ It was suggested that in PAH progression, inhibition of ET-A signaling might relieve the pressure on the pulmonary artery by reducing the vasoconstriction of vascular smooth muscle cells.^93^ Several ET receptor antagonists such as bosentan, ambrisentan and macitentan have already been developed and approved, or are in trial for PAH treatment. Although the inhibition of ET-A activity by ET-A-selective or non-selective agents causes hepatotoxicity during PAH treatment, it provides sustained functional and physiological improvements to the pulmonary artery and relief of PAH symptoms.^93, 94^ It has long been regarded that NO is an important factor in vascular homeostasis. NO is released from the endothelium and modulates pulmonary and systemic vascular smooth muscle tones. Mechanistically, NO can activate soluble guanylate cyclase, resulting in increased levels of intracellular cyclic GMP. It was also reported that NO production is greatly reduced in PAH patients, whereas phosphodiesterase type 5, a major enzyme for cyclic GMP clearance, demonstrated increased expression, suggesting that the inhibition of phosphodiesterase type 5 might reverse PAH progression by relaxation of smooth muscle and inhibition of cell proliferation.^95, 96^ Several lines of phosphodiesterase type 5 inhibitors including sildenafil, tadalafil and vardenafil have been approved for PAH treatment. The improvement of PAH progression by phosphodiesterase type 5 inhibitor therapy has been well evaluated in multiple case reports, along with the tolerated levels of side effects such as headache, sudden visual loss and sudden hearing loss.^97^ Recently, several antiproliferative strategies using tyrosine kinase inhibitors were also developed. Platelet-derived growth factor receptor, epidermal growth factor receptor and fibroblast growth factor signal through tyrosine kinase receptors in the pathological remodeling of the vascular bed and have been targeted by multiple chemical inhibitors including imatinib, nilotinib, gefitinib, sorafenib and sunitinib, for reversal of PAH progression.^98, 99^ Although a greater understanding of the pathogenesis of PAH and PAH-specific therapies has led to significant advances, the disease is still incurable and ultimately fatal. Future therapies need to consider both regulation of pulmonary vascular tone and restoration of pulmonary vascular remodeling, to provide a more effective treatment for PAH through modulation of all the multifactorial factors such as epigenetic changes, pathobiological injurious events and genetic factors affecting PAH development.

## Epigenetic modulation-based therapies for PH

Recent accumulating evidence has suggested that PAH is a complex and multifactorial disorder including overall endothelial malfunction, hyperproliferative vascular cells, anti-apoptosis of multiple compositions of the lung and proinflammatory response in the vascular bed, demonstrating the ‘maladaptive status' of pulmonary arteries. This pathogenic concept gives credit to the environment and epigenetic alterations as a cause of PAH. Overall, it has been proposed that the progression and phenotypic variability of PAH may be affected by multifactorial factors such as genetic background, epigenetic changes, gender and pathobiological injurious events (virus, drug, toxin, hypoxia, inflammation and so on), whereas in a genetically susceptible background the effects of epigenetic changes and/or pathobiological injurious events may aggravate the disease severity^6^ (Figure 1). Interestingly, several experimental improvements through epigenetic modulations have been reported, which should be further evaluated for application to PAH patients. Among the epigenetic mechanisms, miRNAs have the capacity to regulate multiple target genes that may be involved in the pathogenesis of PAH, whereas dysregulation of miRNA expression could lead to the pathogenesis of PAH, which is a representative multifactorial disease. Alterations in the expression patterns of many miRNAs have been reported in PAH. Therefore, strategies to restore aberrant miRNA expression hold potential therapeutic value. Stable, efficient and nontoxic properties of miRNA mimics or inhibitors *in vivo* are an attractive therapeutic approach for the restoration of altered miRNA expression to physiological levels in PAH, while rescuing the expressions of target genes. However, a specific delivery strategy needs to be developed, to allow targeting of the lung vasculature to minimize off-target effects.^1, 35^ Pursuit of the transcriptional mechanisms of miRNA expression may also be a key field of research in PAH. Altered miRNA expression could be restored via modulation of the transcriptional mechanisms of miRNA expression. Several medical research groups are currently testing the therapeutic capability of modulation of HDAC in PAH pathogenesis, which are known to regulate cell proliferation and survival, as the results of both broad spectrum HDAC inhibitors and selective inhibitors demonstrated rescue of experimental PH in a rodent model.^8, 9, 100^ However, broad-spectrum HDAC inhibitors such as trichostatin A have displayed adverse clinical effects such as right ventricular dysfunction and marked induction of cellular apoptosis in coronary artery endothelial cells, indicating that particular attention is needed before their use as therapeutic agents in PAH subjects.^101^ Interestingly, most of the recent studies have indicated that the adverse effects observed could be avoided through selective inhibition of class IIa HDACs, suggesting that class IIa HDACs inhibitors may be a promising new treatment strategy for PAH, while allowing minimization of the side effects.^9^ The exploration of specific functional targets of histone modification and enhancement of selectivity of the chemical inhibitors to histone-modifying enzymes will be needed before application to PAH patients. Lastly, the use of DNMT inhibitors is another option to reverse the progressions of PAH associated with environmental and epigenetic alteration. For example, the down-expressional change of SOD2 was reported in a rat PAH model, which can be reversed by treatment with 5-azacytidine, a DNMT inhibitor. Although, the overall therapeutic concepts regarding epigenetic modulation in PAH are still being tested at the level of experimental conditions, gaining a precise biofunctional understanding of the genetic (for example, miRNAs) and chemical modulators (for example, inhibitors of histone and DNA-modifying enzymes) might open the door for novel treatment of PAH patients. However, careful attention should be given to epigenetic modulation-based therapies for PAH, to ensure their specificity.

## Conclusion

Although epigenetic changes through DNA methylation and histone modification have been well defined in the pathogenesis of many human diseases such as cancer and peripheral hypertension,^44, 102, 103^ their application in the pathogenesis of PAH still remains in critical need of examination. A large amount of the research has been conducted on determining the role of miRNA and other epigenetic mechanisms.^35^ In order to gain a better understanding of the cause of PAH development, the intricate intersecting pathways between histone modification/DNA methylation and miRNA, which can be regulated by miRNA, histone modification or DNA methylation, need to be established. In addition, as epigenetic technology has been advanced to allow DNA methylation analysis, DNA/protein interaction analysis and chromatin accessibility/conformation assays, employment of such methods may allow the complex network of the many genes regulated by epigenetic mechanisms to be uncovered. The application of these epigenetic technologies to PAH research will provide a greatly improved understanding for the development of new drugs via novel targets and signaling pathways associated with PAH, and key insights into potential therapeutic strategies for PAH. Thus, future studies are needed to examine the role of epigenetics in the pathogenesis of PAH, as well as the therapeutic potential in experimental PH models.

## Figures and Tables

**Table 1:** miRNAs involved in the pathogenesis of PAH

*Cell type*	*miRNA*	*Target mRNA*	*Function of miRNA*	*Animal model*	*Reference*
PASMC	miR-130	CDKN1A	Increase of proliferation, no effects on apoptosis	Chronic hypoxia in mice	^104^
PAEC/PASMC	miR-17/20a	BMPR2/PDLIM5, CDKN1A	Increase of proliferation, SMC phenotypic switch	Chronic hypoxia in mice and MCT in rats	^64, 65, 105, 106^
PAEC/PASMC	miR-130/301	PPARγ	Vasoconstriction, increase of proliferation	Chronic hypoxia+SU-5416 in mice, MCT in rats and chronic hypoxia in mice	^107, 108^
PASMC	miR-210	MKP-1, E2F3	Increase of proliferation, inhibition of apoptosis	Chronic hypoxia in mice	^109, 110^
PASMC	miR-451		Increase of migration under serum-free conditions, no effect on proliferation	Chronic hypoxia in mice	^111^
PASMC	miR-193-3p	IGF1R, ALOX5, ALOX12, ALOX15	Inhibition of proliferation	MCT in rats and chronic hypoxia in mice	^112^
PASMC	miR-9		Increase of proliferation, SMC phenotypic switch		^113^
PASMC	miR-190	KCNQ5	Vasoconstriction	Chronic hypoxia in rats	^114^
PAEC	miR-27a	PPARγ	Increase of proliferation	Chronic hypoxia in mice	^115^
Fibroblast	miR-124	PTBP1, MCP-1	Inhibition of proliferation, migration and inflammatory phenotype	Chronic hypoxia in mice and rats, chronic hypoxia+SU-5416 in mice	^36^
PASMC	miR-124	CAMTA1, PTBP1, NFATc1	Inhibition of proliferation, SMC phenotypic switch	Chronic hypoxia in mice	^116^
PASMC	miR-138	MST1	Inhibition of apoptosis		^117^
PAEC	miR-424/503	FGF2, FGFR1	Inhibition of proliferation and migration, cell cycle arrest	MCT in rats and chronic hypoxia+SU-5416 in rats	^43^
PASMC	miR-206	NOTCH-3	Increase of proliferation and migration, inhibition of apoptosis, SMC phenotypic switch	Chronic hypoxia in mice	^118^
PASMC	miR-145	ACE, DAB2, FSCN1	Vascular remodeling	Chronic hypoxia in mice and miR-145 knockout mice	^119^
PASMC	miRNA-328	IGF1R, CaV1.2	Inhibition of proliferation, increase of apoptosis, vasoconstriction	Chronic hypoxia in rats and miR-328 transgenic mice	^120^
PASMC	miR-204	SHP2	Inhibition of proliferation, increase of apoptosis	MCT in rats	^121^

Abbreviations: ACE, angiotensin-converting enzyme; ALOX5, arachidonate 5-lipoxygenase; ALOX12, arachidonate 12-lipoxygenase; ALOX15, arachidonate 15-lipoxygenase; BMPR2, bone morphogenetic protein receptor type II; CAMTA1, calmodulin-binding transcription activator 1; CaV1.2, L-type calcium channel 1C; CDKN1A, cyclin-dependent kinase inhibitor 1A; DAB2, disabled-2; E2F3, transcription factor E2F3; FGF2, fibroblast growth factor 2; FGFR1, fibroblast growth factor 1 receptor; FSCN1, fascin actin-bundling protein 1; IGF1R, insulin growth factor 1 receptor; KCNQ5, potassium voltage-gated channel subfamily KQT member 5 protein; MCP-1, monocyte chemotactic protein-1; MCT, monocrotaline; MKP-1, mitogen-activated protein kinase phosphatase-1; miRNA, microRNA; MST1, serine/threonine kinase 4; MYOCD, myocardin; NFATc1, nuclear factor of activated T-cells, cytoplasmic, calcineurin-dependent 1; NOTCH-3, neurogenic locus notch homolog 3 protein 3; PAEC, pulmonary artery endothelial cell; PAH, pulmonary arterial hypertension; PASMC, pulmonary artery smooth muscle cell; PDLIM5, PDZ and LIM domain protein 5; PPARγ, peroxisome proliferator–activated receptor-γ PTBP1, polypyrimidine tract-binding protein 1; SHP2, Src homology-2 domain containing protein tyrosine phosphatase 2.
